# Ligustilide Prevents Radiation Enteritis by Targeting Gch1/BH_4_/eNOS to Improve Intestinal Ischemia

**DOI:** 10.3389/fphar.2021.629125

**Published:** 2021-04-22

**Authors:** Tao Yan, Shun Guo, Tian Zhang, Zhimin Zhang, An Liu, Song Zhang, Yuan Xu, Yuhong Qi, Weihe Zhao, Qinhui Wang, Lei Shi, Linna Liu

**Affiliations:** ^1^ Department of Pharmacy, The Second Affiliated Hospital of Air Force Medical University, Xi’an, China; ^2^ Department of Cardiology, General Hospital of Xinjiang Military Command, Urumqi, China; ^3^ Department of Radiotherapy, The Second Affiliated Hospital of Air Force Medical University, Xi’an, China

**Keywords:** radiation enteritis, ligustilide, GTP-Cyclohydrolase 1, tetrahydrobiopterin, endothelial nitric oxide synthase

## Abstract

There is a high incidence of radiation enteritis (RE) after abdominal radiotherapy. The occurrence of RE seriously affects the treatment and quality of life of patients; however, its pathogenesis is complex and there are no effective drugs for its prevention or treatment. Intestinal ischemia plays an important role in the occurrence of enteritis. Previous studies have shown that targeting GTP-cyclohydrolase 1 (Gch1) to improve intestinal ischemia could be a new strategy to prevent and treat RE. A high content of the naturally occurring phthalide derivative ligustilide (LIG) has been found in the plant drug Rhizoma Ligustici Chuanxiong for the treatment of cardiovascular diseases. The purpose of this study was to evaluate the protective effects of LIG on RE. Ionizing radiation (IR) rat and endothelial cell models were used to observe and record rat body weights and stool morphologies, measure intestinal blood perfusion by laser Doppler blood flow imaging, determine the diastolic functions of mesenteric arteries, detect the levels of Gch1/BH_4_/eNOS pathway-related proteins and regulatory molecules in the mesenteric arteries and endothelial cells, and predict affinity by molecular docking technology. The results showed that LIG significantly improved the body weights, loose stools, intestinal villi lengths, intestinal perfusion and vasodilatory functions of IR rats. LIG also significantly improved Gch1 protein and BH_4_ levels in the mesenteric arteries and endothelial cells after IR, increased the NO content, reduced superoxide accumulation, and improved p-eNOS (Ser1177) levels in endothelial cells. LIG has good affinity for Gch1, which significantly improves its activity. These results indicate that LIG is the preferred compound for the prevention and treatment of RE by improving intestinal ischemia through the Gch1/BH_4_/eNOS pathway. This study provides a theoretical basis and new research ideas for the development of new drugs for RE.

## Introduction

Radiotherapy is a commonly used cancer treatment method, and approximately 50–60% of cancer patients need to receive radiation therapy. However, during intraperitoneal or pelvic radiotherapy, radiation enteritis (RE) may occur in more than half of patients. The occurrence of RE not only seriously affects the follow-up treatment of patients but also increases the medical burden and may, in severe cases, lead to patient death. However, the pathogenesis of RE is complex, and this process is accompanied by insufficient intestinal villus regeneration, a loss of tight junction proteins, mucosal inflammation and other processes. Currently, there is a lack of effective preventative drugs for RE (Webb et al., 2013; Lu et al., 2019).

The blood supply to tissues and organs is a key factor in maintaining organ function. In a previous study, we found that ionizing radiation (IR) could lead to a significant reduction in the intestinal blood supply, causing mesenteric vascular ischemia and inducing RE. The vascular endothelium is relatively fragile and extremely vulnerable to IR (Soloviev and Kizub, 2019). In our previous studies, we found that targeting the downregulation of GTP-cyclohydrolase 1 caused by IR to prevent endothelial dysfunction could be a new strategy for the prevention of RE (Yan et al., 2020). Gch1 is the rate-limiting enzyme in the synthesis of tetrahydrobiopterin, the content of which plays an important role in maintaining endothelial NOS function, producing NO and maintaining vasodilation (Douglas et al., 2018b; Keszler et al., 2019). However, there is a lack of drugs that can improve this reduction in Gch1 observed after IR.

**FIGURE 1:**
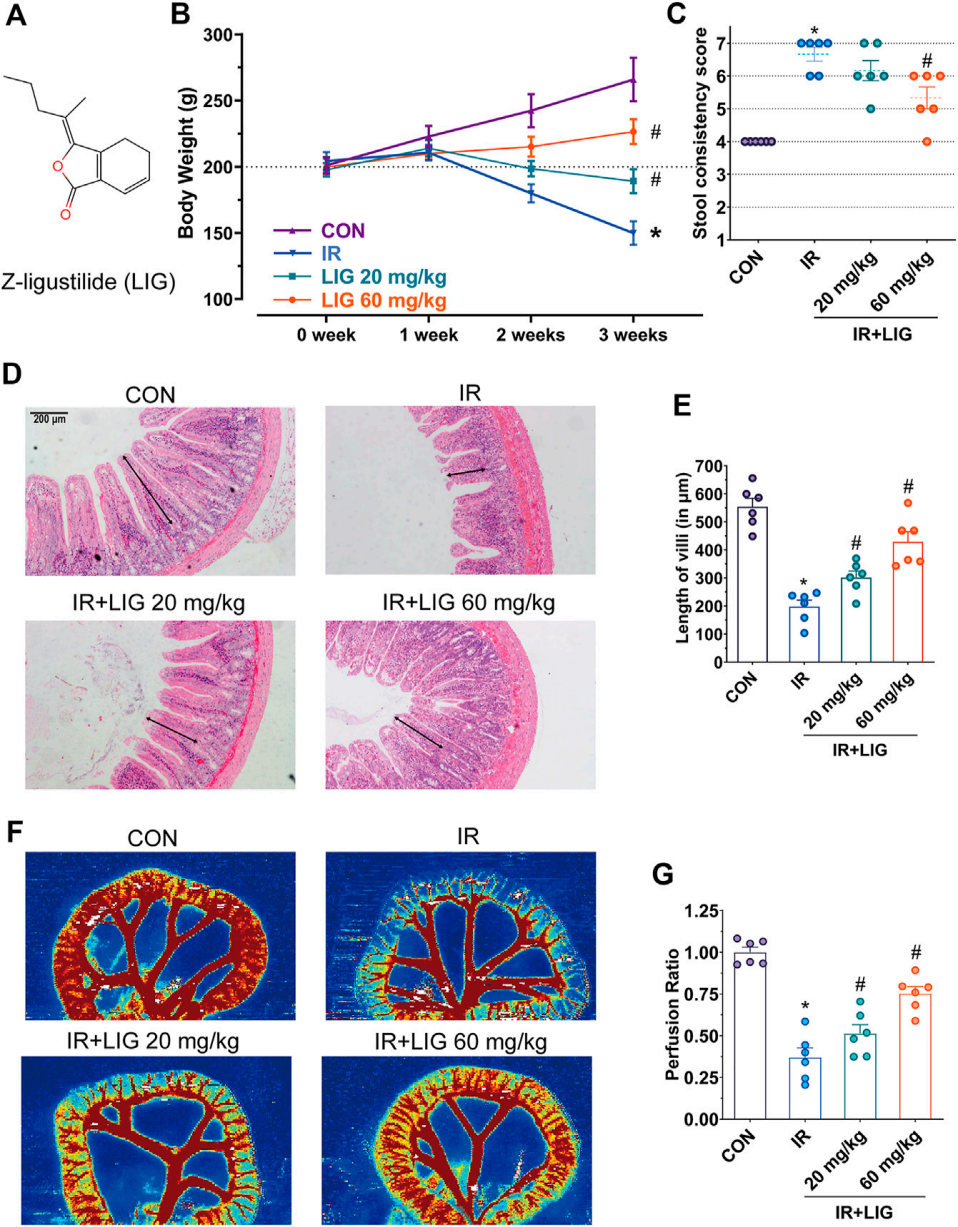
Effects of LIG on RE. **(A)** Molecular structure diagram of LIG. Rats received 4 Gy of IR and intraperitoneal injection of LIG daily (20 or 60 mg/kg). Rat **(B)** body weights and **(C)** stool consistency was recorded weekly. **(D)** Representative histopathological images showing the extent of jejunum damage after 3 weeks of IR with or without LIG (20 or 60 mg/kg, per day) and **(E)** alterations in villus length. **(F)** Representative laser Doppler perfusion images of rat samples after 3 weeks of IR with or without LIG and **(G)** laser Doppler perfusion ratios showing alterations in intestinal perfusion. CON, control group; IR, ionizing radiation; LIG, ligustilide. Data are the mean ± SEM; *n* = 6. *p < 0.05, significantly different from the CON group; ^#^ *p* < 0.05, significantly different from the IR group.

Ligustilide (3-butylidene-4,5-dihydroisodenzofuranone; LIG) is a naturally occurring phthalide derivative that is found in high amounts in herbs (Figure 1A), such as *Ligusticum chuanxiong* Hort (Chuanxiong, CX) and *Angelica sinensis* (Oliv.) Diels (Danggui, DG); its proportion in the volatile oil of CX is more than 40%. These herbs are widely used in the prevention and treatment of cardiovascular diseases in traditional medicine in Southeast Asia, especially in China (Wang et al., 2019). 3-Butyl-1(3H)-isobenzofuranone, with a similar structure to that of LIG, has been approved for clinical use to treat strokes in China. Current studies have found that LIG has anti-inflammatory, tumor growth inhibition, and labor pain pharmacological effects (Xie et al., 2020). Therefore, whether LIG has a protective effect on RE and its subsequent mechanism of action need to be further clarified.

Therefore, we explored whether LIG, a classic cardiovascular therapeutic drug, has a protective effect against RE. The effects of LIG on body weight, stool consistency and intestinal pathology were investigated in IR rats. Laser Doppler and isometric tension vasomotor studies were also performed to examine the effects of LIG on blood perfusion and endothelial relaxation function after IR. The effects of LIG on the Gch1/BH_4_/eNOS pathway were proven by an IR human umbilical vein endothelial cell (HUVEC) model and by virtual docking analyses. This study provides theoretical support that LIG could prevent RE by the targeted improvement of Gch1 and provides new ideas for the research and development of preventative RE drugs.

## Materials and Methods

### Chemicals and Reagents

LIG (purity > 98%) was purchased from Shanghai Yuanye Bio-Technology Co., Ltd. (Shanghai, China). (6 R)-5,6,7,8-Tetrahydrobiopterin dihydrochloride (BH_4_, purity > 98%) was from Shanghai Yuanye Bio-Technology Co., Ltd., Shanghai, China. The BCA Protein Assay Kits were from Beyotime Biotechnology, Shanghai, China) and dihydroethidium (DHE) was from Shanghai Macklin Biochemical Co., Ltd. (Shanghai, China). Antibodies were obtained from the following sources: Gch1 (Sigma-Aldrich, St. Louis, MO, United States), anti-eNOS (phospho-Ser1177, Thr495, Cell Signaling Technology, Danvers, MA, United States), anti-eNOS (Cell Signaling Technology, Danvers, MA, United States) and GAPDH (Abcam, Cambridge, MA, United States). All other chemicals and reagents were of the highest quality available commercially.

### Animals

Male Sprague-Dawley rats (220–250 g, China, RRID: MGI: 5651135) were from the Experimental Animal Center of Air Force Medical University (Xi’an, Shaanxi, China). All animals were housed in a temperature-controlled environment (20°–25°C) and provided access to standard rodent chow and water on a 12-h light-dark schedule. All animal care and experimental procedures were approved by The Second Affiliated Hospital of Air Force Medical University Institutional Review Board according to international and national laws and policies. All efforts were made to minimize the number of animals used and their suffering. All animals were acclimated for at least 7 days before the experiments.

### Irradiation

Rats were irradiated using a linear accelerator (Siemens Primask, Germany) at the Department of Radiotherapy, Second Affiliated Hospital of Air Force Medical University, Xi’an, Shaanxi, China that was adjusted to provide X-ray irradiation. The conventional radiation source was delivered at 400 monitor units/min at a 100 cm source-skin distance. Rats were anesthetized by intraperitoneal injection of pentobarbital sodium (100 mg/kg) and were given a dose of 4 Gy X-ray radiation to the abdomen every day for 21 days, while other parts of the body were shielded by grating technology (Yan et al., 2020).

### Experimental Design

Rats were randomly divided into four groups of six animals each. The control group (CON) received a daily intraperitoneal injection of 0.9% sodium chloride solution for 21 days. The IR group was given an intraperitoneal injection of 0.9% sodium chloride solution daily during irradiation. The LIG + IR groups received LIG (20 or 60 mg/kg body weight) dissolved in 0.9% sodium chloride solution by intraperitoneal injection each day during irradiation (Kuang et al., 2014).

### Rat Body Weights and Stool Consistency Measurements

Rats in each group were weighed weekly before and during IR exposure. After the third week, each animal was put into a separate cage on top of a clean white piece of paper and inspected for diarrheal features for 12 h. During the observation period, a modified Bristol Stool score was used to grade the stool. The numeric rating scale was as follows: 1-Separate hard lumps, like nuts, 2-Sausage-shaped, but lumpy, 3-Like a sausage but with cracks on its surface, 4-Like a sausage or snake, smooth and soft, 5-Soft blobs with clear cut edges, 6-Fluffy pieces with ragged edges, a mushy stool, 7-Watery, no solid pieces, entirely liquid. (Vandeputte et al., 2016).

### Histology

Animals were euthanized after irradiation, and segments of the jejunum and mesenteric arteries were resected. The luminal contents were washed with a 0.9% sodium chloride solution. Segments were pinned on paraffin blocks, floated upside down in 10% buffered formalin overnight at 4°C, dehydrated and then embedded in paraffin. Sections were cut with a Leica sliding microtome (SM 2000R, Nussbach, Germany), and the slides were stained with hematoxylin and eosin. Sections were evaluated quantitatively and qualitatively in a blinded fashion. Selected representative intestinal sections were processed for ultrastructural studies (Xu et al., 2020). The length of the intestinal villi and the blood vessel wall thickness or circumference were calculated by ImageJ software (version 1.6, United States).

### Intestinal Blood Perfusion Measurements

For intestinal blood perfusion measurements, laser Doppler tests were performed during the third week. Based on the Doppler principle, the instrument scanned tissues with a laser beam and collected the backscattered light (Huang et al., 2017). The laser Doppler scanner (MoorLDI2-HR, England) was used with the following parameters: laser, infrared; scan distance, 22 cm; bandwidth, 250 Hz to 15 kHz; and scan speed, 4 ms/pixel. Before the experiments, the rats were fasted for 12 h. After the rats were anesthetized by intraperitoneal injection of pentobarbital sodium (100 mg/kg), laparotomies were performed, and the jejunum was separated. Color-coded images were generated from the spatial distribution of tissue perfusion.

### Organ Bath Assays

Vasodilatory function was analyzed using organ bath assays with a wire myograph system (Multi-Myograph 620 M, Denmark) (Li et al., 2017). Rats were euthanized with a lethal dosage of anesthetic. The rat mesenteric arteries were rapidly excised and placed in cool Krebs-Henseleit buffer (KHB; 119 mM NaCl, 1.2 mM KH_2_PO_4_, 4.7 mM KCl, 2.5 mM CaCl_2_, 1.2 mM MgCl_2_, 25 mM NaHCO_3_, and 11 mM glucose). Segments of the arteries were carefully dissected from the surrounding fat and connective tissue. Aortic rings (approximately 2 mm length) were placed in isolated organ baths containing KHB gassed with 95% O_2_ and 5% CO_2_ at 37 °C. Passive tension-internal circumference was determined by stretching to an internal circumference that was equivalent to 90% of that of the blood vessel under 100 mm Hg transmural pressure. After viability was tested using 45 mM KCl, the aortic rings were precontracted with U46619 (100 nM). The concentration-response relaxation curves were established using cumulative concentrations of acetylcholine (Ach) (1 nM–10 µM).

### Cell Culture and Treatments

HUVECs were donated by Dr. Zhang from the Air Force Medical University. Primary HUVECs are isolated from human umbilical vein and cryopreserved at the end of the primary culture. Each lot of cells is tested using immunohistochemical methods for the presence of von Willebrand factor and CD31 antigen. The uptake of DiI-Ac-LDL is also confirmed. The laboratory tests the cells for the presence of mycoplasma, Hepatitis B, Hepatitis C, and HIV-1 viruses, which were not detected. HUVECs cultured *in vitro* at passages 5 to 10 were used for experiments. HUVECs were cultured in Dulbecco’s modified Eagle’s medium, high-glucose medium containing 10% fetal bovine serum, 100 U/ml penicillin and 100 U/ml streptomycin and incubated at 37°C in 5% CO_2_. When the cells were approximately 80% confluent, they were pretreated with LIG (0.01, 0.1, 1.0, 10.0 μM), 2,4-diamino-6-hydroxypyrimidine (DAHP, 10 mM) or N(ω)-nitro-l-arginine methyl ester (L-NAME, 100 μM) for 12 h, followed by 10 Gy of irradiation (Schmidt et al., 2014).

### Determination of Cell Viability

HUVECs were seeded onto 96-well plates and incubated for 24 h. The cells were then exposed to fluorescent Cell Counting Kit-8 (CCK-8) solution (Japan-Dojindo Laboratories, Japan) for 30 min at 37°C. The fluorescence was measured at *λ*_em_ = 494 nm and *λ*_ex_ = 517 nm with an enzyme-labeled instrument (TECAN Infinite M200 Pro, Männedorf) (Mi et al., 2019). The relative cell viability was calculated as the ratio of the absorbance of each treatment group compared to that of the control group.

### Measurement of Tetrahydrobiopterin

Total BH_4_ levels in tissues or cells were analyzed by a high-performance liquid chromatography (HPLC) (LC-2030; Shimadzu, Japan) instrument with a fluorescence detector (RF-20A; Shimadzu, Japan) according to a previously reported method (Fukushima and Nixon, 1980; Valdés et al., 2017). Briefly, BH_4_ levels were detected using an external standard method using a standard curve established with known concentrations of biopterin. Tissues and cells were lyzed, collected and centrifuged at 12,000 r/min for 10 min at 4°C. One of the sample supernatants was saved to quantify the protein concentration, and the other supernatant was oxidized by acidic or basic iodine solution and then analyzed by HPLC. The following HPLC conditions were used: the column was an Agilent 5TC-C18(2) (Agilent Technologies, United States), column temperature of 25°C, and injection volume of 20 μL. The mobile phase was 5% methanol/95% H_2_O, and the flow rate was 0.9 ml/min. The fluorescence detector conditions were *λ*_em_ = 450 nm and *λ*_ex_ = 350 nm. BH_2_ is oxidized to form biopterin, and under acidic conditions, BH_4_ and BH_2_ are oxidized to biopterin. The difference in biopterin content between the oxidation conditions represents the BH_4_ level.

### Biochemical Analyses

The NO levels in tissues and cell culture supernatants were assayed in triplicate using Total Nitric Oxide Assay Kits (Beyotime Biotechnology, China) to detect nitrite (Zhang et al., 2018). Total protein concentrations of the tissue homogenates and cell lysates were detected by BCA Protein Assay Kits (Beyotime Biotechnology, China) following the manufacturer’s recommendations (Poms et al., 2004).

### Superoxide Production

Tissues were incubated with dihydroethidium (DHE, Macklin Biochemical Co., Ltd., China) for 30 min at 37°C, and excess DHE was removed by washing twice with cold PBS (Fernandes et al., 2017). Then, the tissues were pulverized with a mortar and pestle in liquid nitrogen. The powder was transferred to a tube with a spatula.

HUVECs were washed with PBS after irradiation for 12 h and treated with DHE for 30 min in DMEM (Zhao et al., 2005). The medium was removed, and the HUVECs were washed twice with PBS. The fluorescence was then measured using an Olympus IX53 fluorescence microscope (Olympus, Japan) with an FITC filter and excitation/emission wavelengths of 519 and 590 nm, respectively. Acetonitrile was added to cells before scraping. After centrifugation of the cell or tissue samples, the supernatants were transferred and dried in a vacuum concentrator at room temperature. The precipitated layers were lyzed in lysis buffer (Dulbecco’s PBS with 0.1% Triton X-100, pH 7.4).

The initial mobile phase was added to the above dry samples, and the protein was measured with a BCA Protein Kit. Then, the samples were vortexed and injected into an HPLC instrument with a fluorescence detector (Shimadzu, Japan). HPLC conditions were as follows: an Agilent 5TC-C18(2) column (Agilent Technologies, United States), column temperature of 25°C and injection volume of 20 μL. The mobile phase was 90% H_2_O/10% CH_3_CN (0.1% trifluoroacetic acid), and the flow rate was 0.9 ml/min. The fluorescence detector conditions were *λ*_em_ = 580 nm and *λ*_ex_ = 480 nm.

### Western Blot Analysis

Proteins from tissues and cells were extracted and quantified with BCA Protein Assay Kits. Proteins were separated on SDS-PAGE gels and electrophoresed with molecular weight markers. Protein bands were electrotransferred onto polyvinylidene fluoride membranes (Millipore, United States) using cold transfer buffer. The blots were blocked with blocking buffer, and the membranes were incubated with antibody against Gch1 (1:2,000, Sigma-Aldrich, United States), anti-eNOS (1:2,000, Cell Signaling Technology, United States), anti-phospho-Ser1177/Thr495 eNOS (1:2,000, Cell Signaling Technology, United States), overnight at 4°C, using a GAPDH antibody (1:5,000, Abcam, Cambridge, MA, United States) as a loading control. Membranes were washed using Tris-buffered saline washing buffer and incubated with horseradish peroxidase-conjugated immunoglobulin G. Blots were detected by a chemiluminescence detection system and visualized by enhanced chemiluminescence (Bio-Rad, United States). Densitometry measurements were performed using GelPro software (version 7.0, United States).

### Molecular Docking Studies

The molecular docking analyses of LIG in Gch1 (PDB code: 1WUR) were conducted using AutoDock Vina software. The input files were prepared in the graphic interface AutoDock. Gch1 protein structures were downloaded from the Protein Data Bank. The ligands in the computational study were converted into 3D format. Docking involved the addition of hydrogen atoms to the protein, the assignment of bond orders, and the deletion of unnecessary water molecules. The Receptor Grid Generation Workflow was used to define a grid around the bound cocrystallized ligand, and the grid was then used for docking LIG into the ligand binding site. The ligand interaction tool was used to view the interaction of the ligands with the residues at the active site of the Gch1 protein (Lohning et al., 2017).

### GTP Cyclohydrolase 1 Activity Assays

Gch1 activity was determined as described previously (Nar et al., 1995). Briefly, the formation of dihydroneopterin triphosphate and the product of Gch1 were analyzed. The fused or free Gch1 protein was incubated in the dark with GTP assay buffer. Oxidation of dihydroneopterin triphosphate was performed with hydrogen chloride and potassium iodide/iodine in the dark. Samples were decolorized with ascorbic acid, vortexed, neutralized with sodium hydroxide and dephosphorylated with alkaline phosphatase. Samples were then centrifuged, and neopterin was quantified with an enzyme-labeled instrument (TECAN Infinite M200 Pro, Männedorf) with fluorescence detection.

### Statistical Analysis

Data are expressed as the mean ± standard error of the mean (SEM). Significant differences between groups was determined by one-way analysis of variance (ANOVA). After analysis by ANOVA, Bonferroni’s correction was conducted for post hoc t-tests to exclude false positives. Statistical analysis was conducted, and figures were generated with GraphPad Prism (version 8.0, GraphPad Software, Inc., United States). For all tests, *p* < 0.05 was considered statistically significant.

## Results

### Effect of Ligustilide on Stool Consistency and Body Weights of Ionizing Radiation Rats

Adult SD rats with body weights ranging from 180 to 220 g were divided equally into four groups with free access to food and clean drinking water, and the weights of rats in each group were measured every week. The results showed that the weights of the rats in the control group increased by approximately 20 g per week. In the IR group, the weights of rats were lower than the initial weight in the second week. In the third week, the weights of the rats were reduced by approximately 43% compared with the control group, showing a significant difference. The oral bioavailability of LIG is low. According to previous reports, rats received intraperitoneal injections of LIG (20 and 60 mg/kg) every day during the IR period (Xie et al., 2020). Compared with the IR group, the weights of rats increased significantly (Figure 1B).

In the third week, the rat stool consistency was evaluated, and compared with the control group, the diarrhea score of the rats in the IR group increased significantly, and approximately 67% of the rats had liquid stools. Compared with the IR group, the diarrhea score of rats in the 60 mg/kg LIG pretreatment group was significantly lower (Figure 1C). The above results suggest that LIG intervention could significantly improve rat weight loss and diarrhea caused by radiation.

### Ligustilide Improved the Histopathological and Intestinal Blood Perfusion Changes Induced by Ionizing Radiation

Intestinal villi play an important role in the intestinal absorption of nutrients. The pathological results of the jejunum in rats exposed to IR for three weeks showed that the villus length was reduced to 40% of that of the control group (CON, 553.7 ± 74.6/μm, vs. IR, 197.8 ± 56.0/μm). Intraperitoneal injection of LIG during IR significantly increased villus length compared to the IR group (LIG 20, 301.5 ± 56.5/μm, LIG 60, 428.4 ± 88.1/μm) (Figures 1D,E).

A decrease in intestinal perfusion could induce the occurrence of enteritis. Compared with the control group, intestinal blood perfusion decreased significantly after three weeks of IR, according to the results of intestinal flow laser Doppler analysis. Compared with the IR group, the LIG group showed significantly improved intestinal perfusion. These results illustrated that LIG could improve intestinal pathological damage and reduce the decrease in intestinal blood perfusion caused by IR (Figures 1F,G).

### Ligustilide Inhibits the Remodeling of Ionizing Radiation Rat Mesenteric Arteries

**FIGURE 2:**
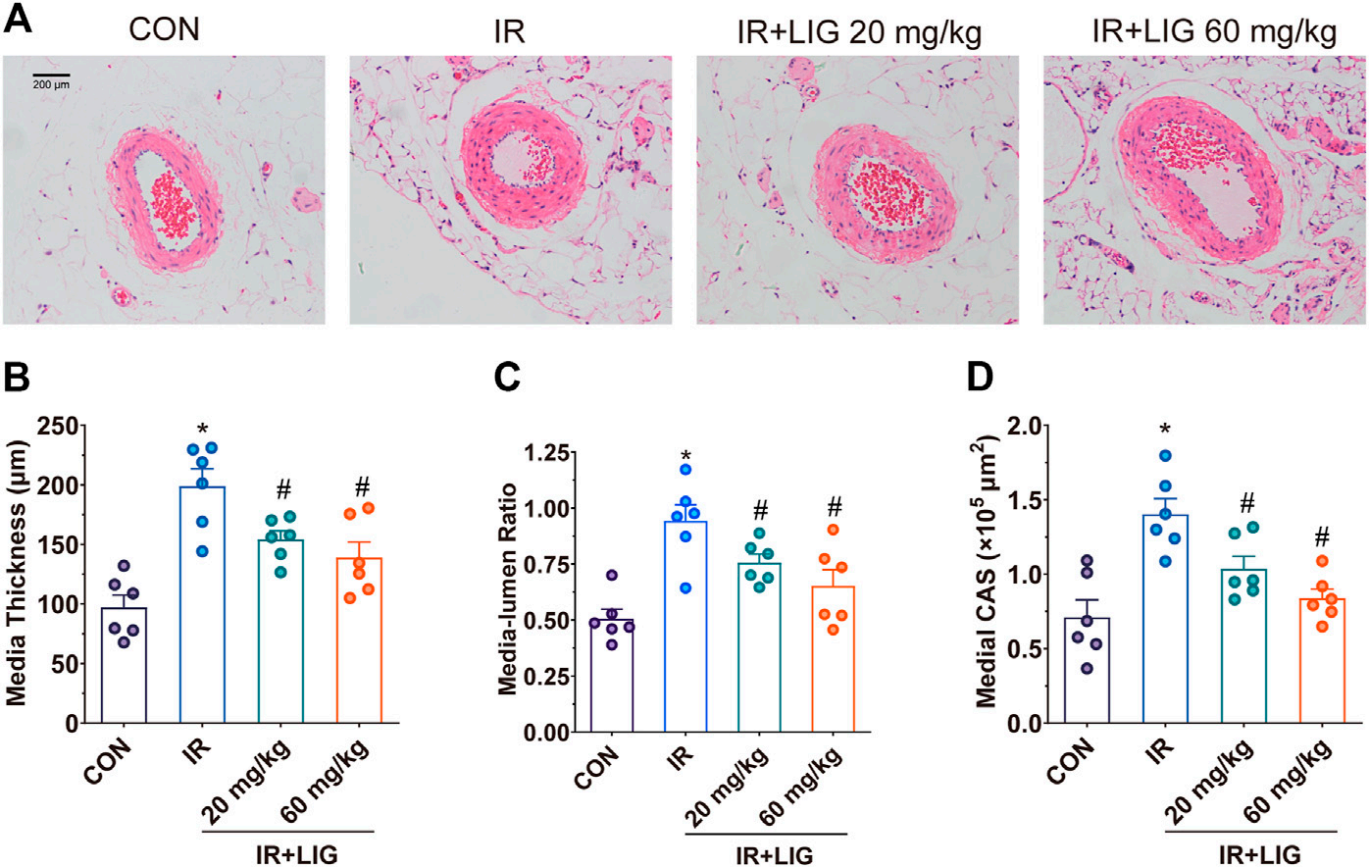
Effects of LIG on mesenteric artery remodeling in rats after IR. **(A)** Representative hematoxylin-eosin staining histopathological images after 3 weeks of IR. Vascular remodeling was evaluated by **(B)** media thickness, **(C)** media-lumen ratio, and **(D)** medial CSA. CSA, medial cross-sectional area. Data are the mean ± SEM; *n* = 6. *p < 0.05, significantly different from the CON group; ^#^ *p* < 0.05, significantly different from the IR group.

Morphological studies showed that the mesenteric arteries of IR rats presented significant changes relative to the control group at 3 weeks. IR resulted in a dramatic increase in the media thickness and media-to-lumen ratio. The medial cross-sectional area (CSA) was also significantly enhanced in IR rats, a typical feature of hypertrophic remodeling. Vascular structure alterations were significantly reduced after treatment with LIG (Figure 2).

### Ligustilide Increased GTP Cyclohydrolase 1 Protein Levels, Improved Tetrahydrobiopterin Deficiency and Vascular Relaxation, and Increased NO Generation in Ionizing Radiation Rats

**FIGURE 3:**
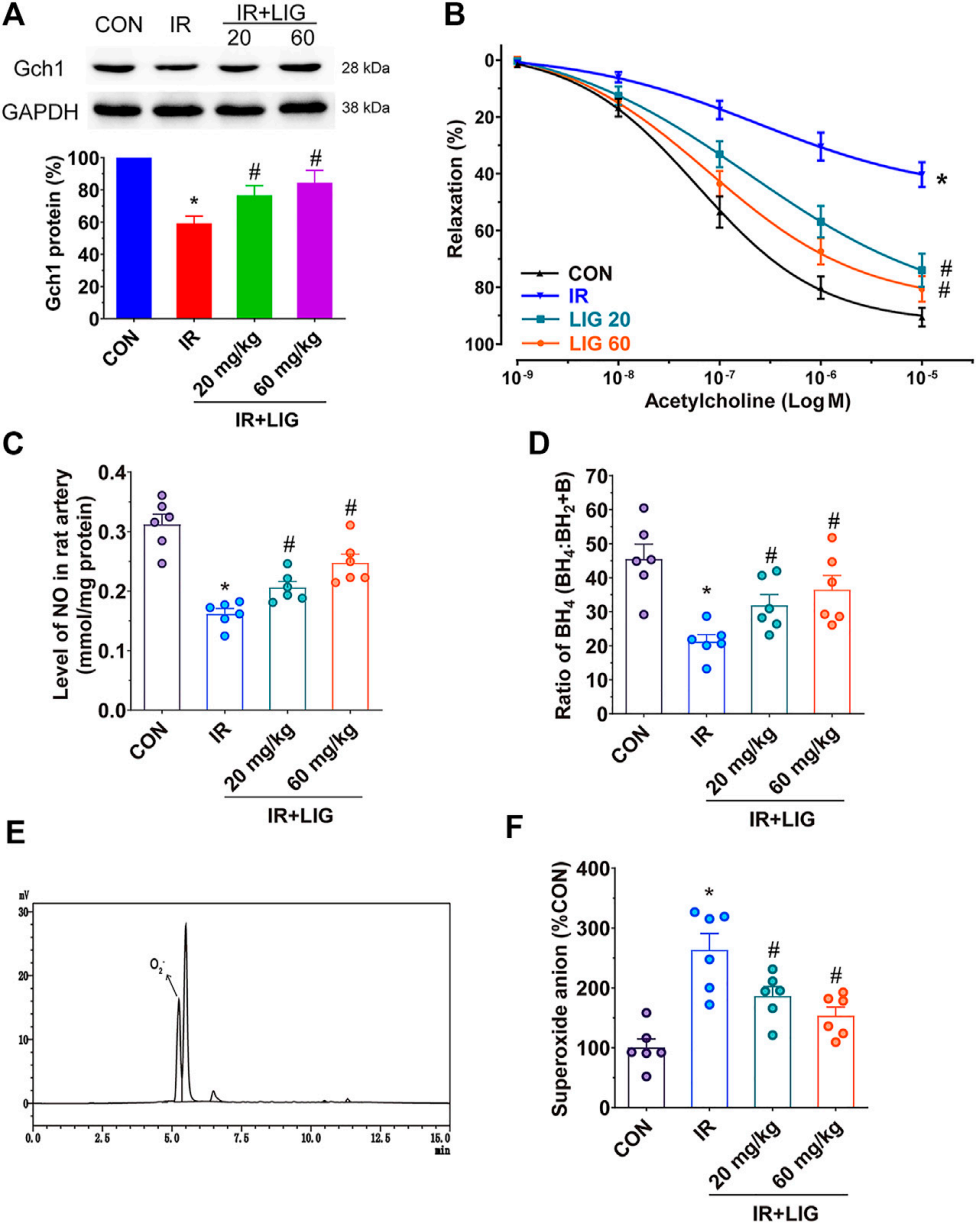
Effects of LIG on Gch1 protein levels, vasodilatory functions and BH_4_ levels in IR-exposed rat mesenteric arteries. Rats received 4 Gy of IR and intraperitoneal injection of LIG daily for 3 weeks (20 or 60 mg/kg). **(A)** Representative immunoblots and quantification as band densities showing phosphorylation of Gch1 with GAPDH as a loading control in rat mesenteric arteries (*n* = 4). **(B)** Receptor-mediated endothelium-dependent vasodilation after acetylcholine treatment. Aortic rings were precontracted with U46619 (100 nM). **(C)** The total amount of NO in the mesenteric arteries was determined by a total NO assay kit. **(D)** Ratio of BH_4_ to BH_2_+B in rat mesenteric arteries. **(E)** Representative chromatogram of superoxide anions detected by HPLC coupled with fluorescence detection in rat mesenteric arteries. **(F)** Relative quantitation of superoxide anions in rat mesenteric arteries. Data are the mean ± SEM; *n* = 6. *p < 0.05, significantly different from the CON group; ^#^ *p* < 0.05, significantly different from the IR group.

We determined the effects of LIG on Gch1 protein levels in IR rat mesenteric arteries. Compared with the control group, IR significantly reduced the level of the Gch1 protein. LIG improved the reduction in Gch1 levels induced by IR (Figure 3A).

Vasodilation is an important factor in maintaining blood perfusion. Through analysis of isometric tension vasomotor studies to determine the sensitivity of the endothelium dependent vasodilator acetylcholine in pre-contracted rat mesenteric small arteries. We found that IR could significantly reduce the vasodilatory functions of the rat mesenteric arteries compared with the control group. After LIG intervention, the vascular relaxation functions of the rats in the two LIG treatment groups significantly improved (Figure 3B).

NO production plays an important role in vasodilation. Measurement of the NO content in the mesenteric artery showed that IR could significantly reduce the NO content compared with the control group (mean CON, 0.3124 ± 0.0168/mmol/mg vs. IR, 0.1620 ± 0.0086/mmol/mg). Compared with the IR group, LIG intervention significantly increased the NO content (mean LIG 20, 0.0261 ± 0.0099/mmol/mg, LIG 60, 0.2474 ± 0.0148/mmol/mg). These results indicated that LIG can improve both the vasodilatory functions and NO content reduced by IR (Figure 3C).

Further detection of Gch1-produced BH_4_ in rat mesenteric arteries revealed that IR could significantly reduce BH_4_ levels compared with the control group. However, LIG significantly increased BH_4_ levels compared to the IR group (Figure 3D).

A BH_4_ deficiency would result in the accumulation of superoxide anions. The superoxide anion contents in the rat mesenteric arteries could be accurately analyzed by reversed-phase (RF)-HPLC, and the sample detection diagram is shown in Figure 3E. The results showed that LIG significantly reduced the accumulation of superoxide anions caused by IR (Figure 3F).

### Ligustilide Increased Cell Viability, Tetrahydrobiopterin, and NO Production, and Decreased Superoxide Anion Accumulation in Ionizing Radiation-Treated HUVECs

**FIGURE 4:**
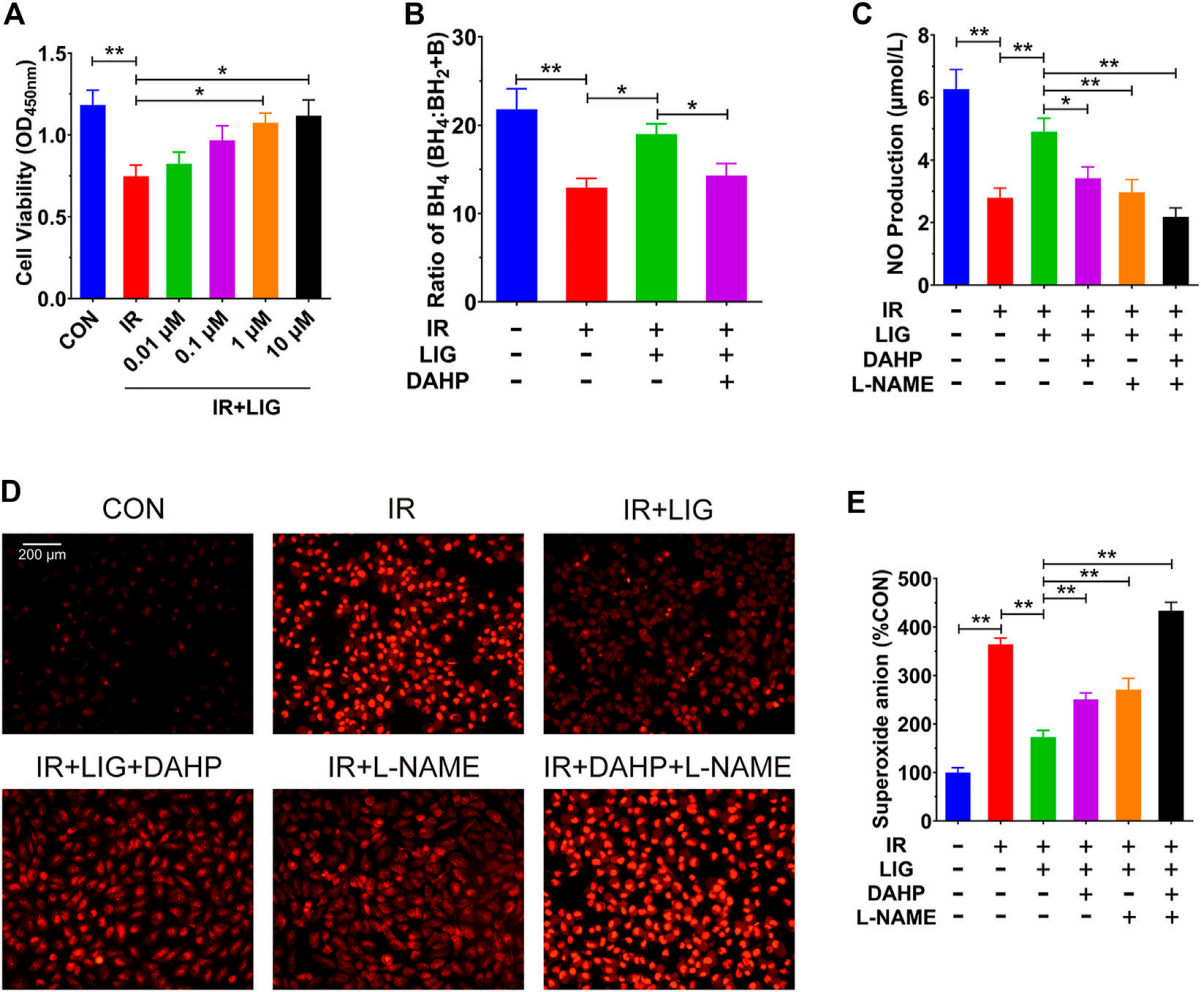
LIG improved endothelial dysfunction in IR-exposed HUVECs. HUVECs were pretreated with LIG (0.01–10.0 μM), DAHP (10 mM) or L-NAME (100 μM) for 12 h followed by 10 Gy of irradiation. **(A)** Quantification of HUVEC cell viability after exposure to IR with or without LIG determined using a CCK-8 kit. **(B)** Ratio of BH4 to BH2+B in HUVECs. **(C)** The total amount of NO in HUVECs was determined by a total NO assay kit. **(D)** DHE fluorescent probe labeling of superoxide anions in HUVECs. **(E)** Quantification of superoxide anions in HUVECs. The concentration of superoxide anions in HUVECs was analyzed using HPLC. DAHP, 2,4-diamino-6-hydroxypyrimidine; l-NAME, N(ω)-nitro-L-arginine methyl ester. Data are the mean ± SEM, *n* = 6. *p < 0.05; **p < 0.01.

To investigate the protective effects of LIG on HUVECs, we pretreated HUVECs with different doses of LIG for 12 h, treated the cells with 10 Gy of IR, and detected cell viability by CCK-8 assay after 12 h (Choi et al., 2018; Yan et al., 2020). The results showed that compared with the control group, IR could significantly reduce cell viability, and 1 and 10 μM LIG could significantly increase the reduction in cell viability caused by IR. There was no significant difference in the improvement in cell viability after IR between the 1 and 10 μM LIG groups; therefore, 1 μM LIG was used as the dose in subsequent experiments (Figure 4A).

In a previous rat experiment, we found that LIG could improve the reduction in the BH_4_ ratio in the mesenteric artery caused by IR. We further examined the effects of LIG on the BH_4_ ratio in HUVECs, and the results showed that IR significantly reduced the BH_4_ ratio compared to the control group. After treatment with LIG (1 μM for 6 h), the BH_4_ ratio was significantly higher than that in the IR group. Cotreatment with the Gch1 inhibitor DAHP (10 mM) and LIG resulted in a significant reduction in the BH_4_ ratio compared to the LIG group (Figure 4B).

We further investigated the effects of LIG on NO content in HUVECs treated with IR. The results showed that LIG could significantly improve the IR-induced reduction in NO in HUVECs. Cotreatment of LIG with either the Gch1 inhibitor DAHP or the eNOS inhibitor L-NAME (100 μM) resulted in significant reductions in NO content in HUVECs compared to the LIG group (Figure 4C).

The results for the superoxide anions in cells also showed that in HUVECs, LIG significantly reduced the accumulation of superoxide anions due to IR. LIG pretreatment with DAHP or L-NAME significantly increased the accumulation of superoxide anions in HUVECs compared to the LIG group (Figures 4D,E). All of the above results demonstrated that LIG can improve HUVEC dysfunction induced by IR, and both Gch1 and eNOS inhibitors can reduce the improvement effects from LIG.

### Effects of Ligustilide on the GTP Cyclohydrolase 1 Protein and Endothelial Nitric Oxide Synthase Phosphorylation in Ionizing Radiation-Exposed HUVECs

**FIGURE 5:**
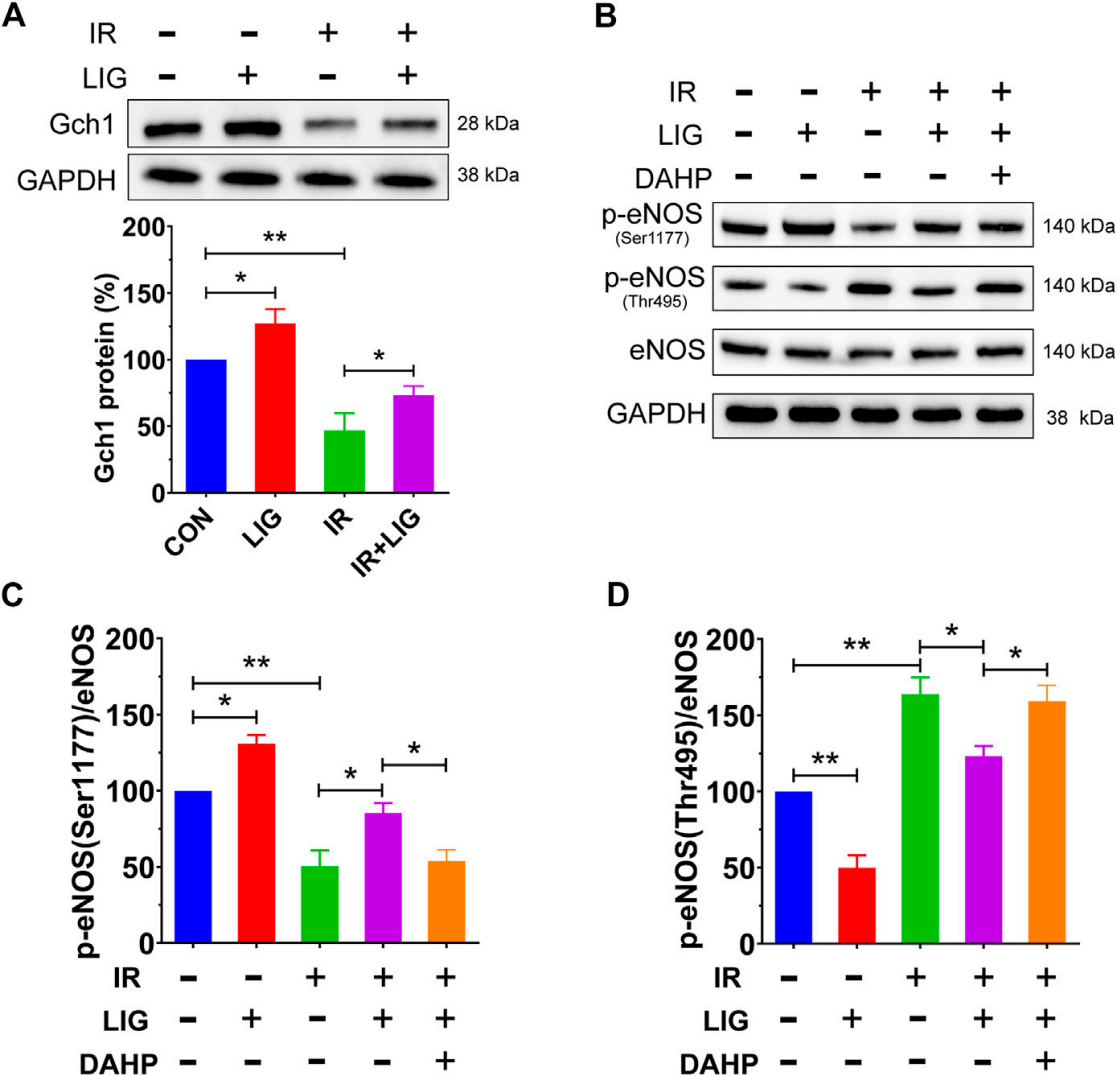
Effects of LIG on the Gch1 protein and phosphorylated eNOS levels in IR-exposed HUVECs. HUVECs were pretreated with LIG (1.0 μM), DAHP (10 mM) or l-NAME (100 μM) for 12 h, followed by 10 Gy of irradiation. **(A)** Representative immunoblots and quantification of band densities showing Gch1 with GAPDH as a loading control after IR of HUVECs. **(B–D)** The levels of *p*-eNOS (Ser1177), p-eNOS (Thr495), and total eNOS protein were measured by western blotting. Representative images of experiments and densitometric analysis of the phosphorylated proteins normalized to the total protein level. Data are the mean ± SEM, *n* = 4. *p < 0.05; **p < 0.01.

The effects of LIG on Gch1 protein levels in HUVECs were investigated by western blot, and the results showed that LIG could significantly improve the protein level of Gch1 compared with the control group. Compared to the IR group, LIG significantly improved the Gch1 reduction due to IR (Figure 5A).

Phosphorylation of eNOS, which is downstream of Gch1, plays an important role in the production of NO, and the results showed that LIG could significantly increase the level of p-eNOS (Ser1177) and decrease the level of p-eNOS (Thr495) compared with the control group. Compared with the IR group, LIG attenuated the decrease in p-eNOS (Ser1177) and increase in p-eNOS (Thr495) induced by IR, while the Gch1 inhibitor DAHP significantly reduced the improvement observed after LIG treatment (Figures 4B–D). The above results suggested that LIG could improve the reduction in the Gch1 protein level in HUVECs induced by IR and further improve the level downstream p-eNOS (Ser1177).

### Ligustilide has Significant Affinity for GTP Cyclohydrolase 1 and Improves GTP Cyclohydrolase 1 Activity

**FIGURE 6:**
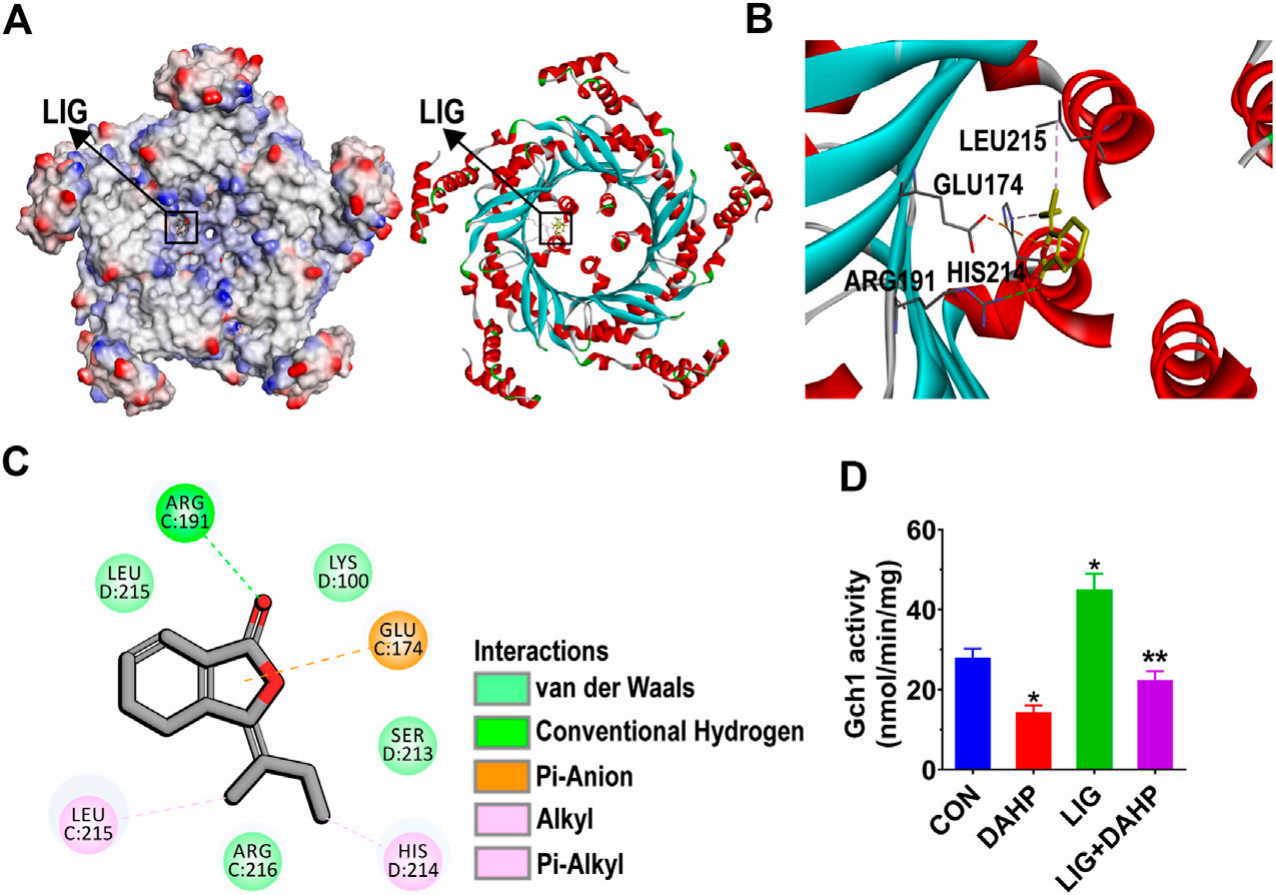
Structural interactions of LIG with Gch1 and their effects on activity. **(A)** Surface of Gch1 colored by ionizability. The black rectangle marks LIG. **(B)** Structural representation of LIG binding to Gch1 (PDB: 1WUR) as inferred from docking simulations. LIG is shown as yellow sticks. **(C)** 2D diagram of the interaction between LIG and Gch1 showing the major binding sites and bonding forces. **(D)** Gch1 activity quantified by fluorescence detection. The fused or free Gch1 protein was incubated in the dark with GTP assay buffer (310 μM). DAHP was added at a concentration of 1 mM. Data are the mean ± SEM, *n* = 6. *p < 0.05, significantly different from the CON group, **p < 0.05, significantly different from the LIG group.

To further investigate the potential mechanism by which LIG regulates Gch1, we conducted molecular docking analysis of LIG. LIG was docked into Gch1 by means of the CDOCKER module of Discovery Studio (Accelrys Inc., San Diego, CA, United States). The binding site was defined from receptor cavities. The Gch1 pentamer forms five similar binding pockets, each of which bind well to LIG. The interactions between Gch1 and LIG involve hydrogen bonds, hydrophobic contacts and pi-anion stacking. The carbonyl group of LIG forms a conventional hydrogen bond with Arg191 of Gch1. The aromatic ring on LIG has a strong pi-anion interaction with the carboxyl group of Glu174. Moreover, LIG is further stabilized in the pocket through hydrophobic contacts with His214 and Leu215 (Figures 6A–C).

The molecular docking results indicated that LIG has significant affinity for Gch1. To further verify whether LIG could improve the activity of Gch1, as reported in the literature, the activity of Gch1 after coincubation with DAHP and LIG was determined. The results showed that LIG could significantly improve the catalytic activity of Gch1, and DAHP could weaken the catalytic effects of LIG (Figure 6D).

## Discussion

As a common complication of clinical radiotherapy, the prevention RE shows greater promise than its treatment, but there is still a lack of effective preventative drugs. Studies have shown that the Gch1/BH_4_/eNOS axis plays an important role in maintaining the intestinal blood supply and preventing RE. However, there is no drug that targets this pathway for the treatment of RE. Our study mainly revealed the following findings. First, LIG significantly improved body weight loss, loose stools, intestinal villus loss, decreased intestinal perfusion and impaired vasodilatory functions caused by IR in rats. Second, LIG could significantly improve the reduction in Gch1 protein levels in rat mesenteric arteries and HUVECs induced by IR, increase the contents of BH_4_ and NO, and reduce the accumulation of superoxide anions. LIG also increased the level of p-eNOS (Ser1177) in HUVECs. Finally, LIG has a strong affinity for Gch1, which can increase its activity.

The occurrence of RE leads to nausea, vomiting, abdominal pain, diarrhea and other symptoms in patients, aggravating the patient's condition. Moreover, severe complications, such as sepsis and multiple organ dysfunction syndrome, can occur (Chater et al., 2019). Therefore, many patients must reduce the radiation dose or terminate radiation treatment. The pathogenesis of enteritis is complex, and there is currently no recognized effective drug for the prevention or treatment of RE (Stacey and Green, 2014). The early drugs for the prevention and treatment of RE mainly eliminated the free radicals produced by IR, such as thiol compounds, but due to their high toxicity, they were quickly eliminated from clinical practice. At present, there are only symptomatic and supportive drugs, such as glutamine, that promote the repair of intestinal epithelial cells, but their effectiveness is still controversial (Vidal-Casariego et al., 2015). Cytoprotective drugs, such as amifostine, show obvious adverse reactions such as vomiting and nausea in clinical use, resulting in poor patient compliance, and thus they are rarely used in clinical practice (Gula et al., 2013).

With deepening study on RE, researchers have found that blood vessels, as highly differentiated organs, are easily damaged by IR exposure during radiotherapy, resulting in vascular endothelial dysfunction, which is an important factor in the induction of enteritis (Panés and Granger, 1998; Leonetti et al., 2020). In our previous study, we found that the Gch1/BH_4_/eNOS axis plays an important role in the prevention and treatment of RE. For instance, the plasma BH_4_ levels in radiotherapy patients is significantly reduced. By targeting and improving the reduction in Gch1 levels after IR and increasing the BH_4_ content, RE can be significantly improved (Yan et al., 2020). However, BH_4_ is very easily oxidized into dihydrobiopterin. Direct supplementation with BH_4_ is not only expensive but also has severe adverse reactions. Currently, it is only used in small amounts for the rare disease phenylketonuria (Flydal et al., 2019; Evers et al., 2020). Therefore, it is of great significance to find new compounds that target the improvement in Gch1 levels for the prevention and treatment of RE.

There is a high LIG content in *Ligusticum chuanxiong* Hort and *Angelica sinensis*. This compound has kidney protection, antispasmodic, anticancer, anti-apoptosis and anti-infectious effects. Currently, no reports have been found regarding the improvement in endothelial dysfunction or the prevention and treatment of RE by targeting Gch1 (Chen et al., 2018; Guo et al., 2020). Given that LIG is the main component of the Chuanxiong Rhizoma and Angelica sinensis medicinal plants, which are widely used for the prevention and treatment of cardiovascular diseases in Southeast Asia and especially China (Peng et al., 2009), we assumed that LIG might prevent RE by targeting Gch1 to prevent the endothelial dysfunction caused by IR. We verified our hypothesis by using previously established IR rat and HUVEC models.

The occurrence of RE can cause weight loss and diarrhea in patients (Zachariah et al., 1997). This study adopted a rat model with continuous low-dose IR, which can better simulate the pathological process of clinical RE (Zhang et al., 2019). Weekly body weight measurements showed that intervention with both 20 and 60 mg/kg LIG significantly improved body weight loss due to IR in rats. During the third week of IR, rat stool consistency and jejunal pathology were examined, and the results showed that 60 mg/kg LIG significantly improved the stool consistency and absence of intestinal villi in rats. Intestinal ischemia is a key factor in the occurrence and development of enteritis. We further investigated the effects of LIG on intestinal blood supply. The laser Doppler blood flow results showed that the two doses of LIG could significantly improve the reduction in intestinal blood supply caused by IR. This reduction in intestinal blood supply may be due to decreased diastolic function caused by vascular remodeling. We evaluated the effects of LIG on mesenteric artery vascular remodeling, and the results showed that LIG could significantly reduce the increase in media thickness, media-to-lumen ratio, and medial CSA induced by IR, indicating that LIG intervention could inhibit the occurrence of vascular remodeling induced by IR.

We have demonstrated that LIG can improve the intestinal blood supply reduction caused by IR. Further, animal models were used to verify whether LIG exerted an improvement by targeting Gch1. The western blot results of the mesenteric artery showed that 20 and 60 mg/kg LIG could significantly improve the reduction in Gch1 protein levels caused by IR. A decrease in Gch1 levels results in an insufficient BH_4_ level, a decrease in the eNOS-catalyzed conversion of L-arginine to L-citrulline, a decrease in NO production, accumulation of superoxide anions, and a decrease in vasodilatory function, finally leading to intestinal hypoperfusion (Chuaiphichai et al., 2017; Douglas et al., 2018a). Rat mesenteric arteries do not show spontaneous tone, it is necessary to first induce a contraction to be able to observe the relaxation to acetylcholine. Acetylcholine causes relaxation of rat mesenteric small arteries by activating of muscarinic M3 receptors at the endothelial cell layer leading to release of NO (Wilson et al., 2016). Our results showed that both doses of LIG could improve the vasodilatory function after IR, increase the contents of NO and BH_4_, and reduce the accumulation of superoxide anions, indicating that LIG could play a protective role by targeting Gch1.

The animal model results showed that LIG could target Gch1 and play a protective role by improving endothelial dysfunction caused by IR. Therefore, we used endothelial cells to verify whether LIG acts through the Gch1/BH_4_/eNOS axis. We first added 0.01, 0.1, 1, and 10 μM LIG to HUVECs 12 h before receiving IR for intervention, and then detected the cell viability using a CCK-8 assay. The results showed that 1 and 10 μM LIG pretreatment could significantly improve the cell viability decline caused by IR. Because there was no significant difference in the improvement in HUVEC viability between the 1 and 10 μM LIG groups, we chose 1 μM LIG to investigate its effects on the Gch1/BH_4_/eNOS pathway after IR. These results showed that LIG could significantly improve the reduction in cellular Gch1 protein levels after IR, increase the BH_4_ and NO contents, and reduce the accumulation of superoxide anions. In its inactive state, eNOS is phosphorylated on Thr495 and forms a complex. The western blot results suggested that LIG could reduce the protein level of p-eNOS (Thr495) and increase the protein level of p-eNOS (Ser1177). We coadministered the Gch1 inhibitor DAHP with LIG to treat HUVECs, and the results showed that administration of the Gch1 inhibitor weakened the LIG-mediated improvement observed in HUVECs. In computer-based molecular docking, molecular simulation software can be used to analyze the structural properties of binding sites, such as the electrostatic field, hydrophobic field, and hydrogen bonding interactions. A database is then used to identify the extent to which the obtained molecular shape and physical and chemical properties match the receptor interaction site and to test the biological activity of these molecules (Saikia and Bordoloi, 2019). The docking results in our analysis suggested that LIG had good affinity for Gch1. We further tested the catalytic activity of LIG on Gch1, and the results further supported that Gch1 was activated by LIG. All of the above results suggested that LIG could play a protective role in HUVECs exposed to IR through the Gch1/BH_4_/eNOS pathway.

**FIGURE 7:**
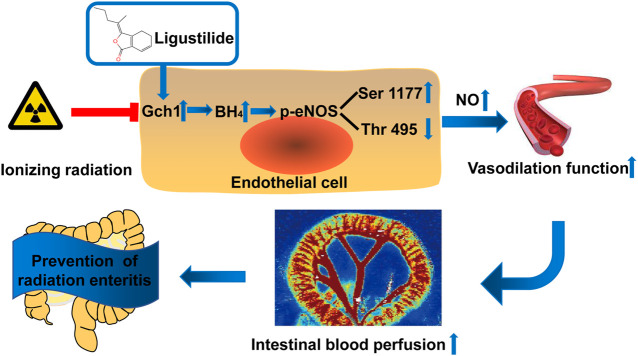
Schematic illustration. Ligustilide prevents radiation enteritis by targeting to improve the levels of Gch1 and eNOS phosphorylated s1177 proteins in vascular endothelial cells after ionizing radiation, increase the production of BH_4_ and NO, correct vascular diastolic function, and maintain intestinal blood perfusion.

In conclusion, in this study, we found that LIG can be used as a preferred compound for the prevention and treatment of RE, which was verified by animal and cell models. The mechanism of action is through targeted improvements in the Gch1 and eNOS phosphorylation protein levels in the vascular endothelium after IR, an increase in the content of NO generated by BH_4_, correction of vascular diastolic function, and maintenance of intestinal blood supply (Figure 7). This study has provided a new theoretical basis for the research and development of drugs for RE and offered new ideas for the research of new drug targets.

## Data Availability

The original contributions presented in the study are included in the article/Supplementary Material, further inquiries can be directed to the corresponding authors.

## Abbreviations

RE, radiation enteritis; IR, ionizing radiation; BH_4_, tetrahydrobiopterin; eNOS, endothelial nitric oxide synthase; Gch1, GTP cyclohydrolase 1; LIG, ligustilide; CSA, cross-sectional area; DAHP, 2,4-diamino-6-hydroxypyrimidine; L-NAME, N(ω)-nitro-L-arginine methyl ester; HUVECs, human umbilical vein endothelium cells; DHE, dihydroethidium.
